# Decreased digital skin microvascular blood flow before and after cold stimulus using Laser Doppler imaging in juvenile systemic sclerosis

**DOI:** 10.1186/1546-0096-9-S1-P76

**Published:** 2011-09-14

**Authors:** Maria Teresa RATerreri, Daniela Gerent Petry Piotto, Marcelo José Uchôa Corrêa, Cristiane Kayser, Claudio Len

## Background

Laser Doppler imaging (LDI) is a new method for assessing the functional superficial skin blood flow in systemic sclerosis and Raynaud's phenomenon.

## Aim

To evaluate the dynamic behavior of fingertip microvascular blood flow before and after cold stimulus, using LDI in children and adolescents with Raynaud's phenomenon secondary to juvenile systemic sclerosis (JSS), primary Raynaud's phenomenon and healthy controls, and to compare it with structural microvascular abnormalities evaluated by nailfold capillaroscopy.

## Methods

We included 5 patients with JSS, 5 children and adolescents with primary Raynaud's phenomenon and 5 healthy controls matched for gender and age. All subjects were submitted to nailfold capillaroscopy. The fingertip blood flow (FBF) from the 4 fingertips of left hand was measured using the LDI at baseline, at 1, 4, 10, 25 and 40 minutes after cold stimulus.

## Results

Mean baseline FBF was significantly lower in JSS patients when compared to controls (204.5 ± 210.1 vs 649.6 ± 92.5 PU, p <0.002). There was also a trend towards lower mean baseline FBF in patients with JSS compared to patients with primary Raynaud's phenomenon. We observed a sharp drop in average FBF 1 minute after the cold stimulus in all groups. The mean FBF remained significantly lower in JSS patients when compared to the other groups in all measurements. Moreover, a blood flow recovery in the follow-up was not observed 27 minutes after cold stimulus in JSS patients. All JSS patients presented scleroderma pattern in nailfold capillaroscopy with fewer number of capillaries, higher number of enlarged capillaries and deletion score compared with the other two groups (p=0.009, p=0.025, p=0.001, respectively).

## Conclusion

In JSS patients LDI showed a lower superficial blood flow in the fingertips before and after cold stimulus compared to healthy controls and patients with Raynaud's phenomenon. Quantification of blood flow by LDI can be useful for disease follow-up and for monitoring therapeutic interventions in JSS patients.

